# Acute cardiovascular events in patients with community acquired pneumonia: results from the observational prospective FADOI-ICECAP study

**DOI:** 10.1186/s12879-021-05781-w

**Published:** 2021-01-25

**Authors:** Filippo Pieralli, Vieri Vannucchi, Carlo Nozzoli, Giuseppe Augello, Francesco Dentali, Giulia De Marzi, Generoso Uomo, Filippo Risaliti, Laura Morbidoni, Antonino Mazzone, Claudio Santini, Daniela Tirotta, Francesco Corradi, Riccardo Gerloni, Paola Gnerre, Gualberto Gussoni, Antonella Valerio, Mauro Campanini, Dario Manfellotto, Andrea Fontanella, for the FADOI-ICECAP Study Group, T. Attardo, G. Augello, F. Dentali, L. Tavecchia, V. Gessi, F. Pieralli, G. De Marzi, A. Torrigiani, L. Corbo, G. Uomo, F. Gallucci, C. Mastrobuoni, F. Risaliti, A. Giani, L. Morbidoni, Consalvo Teodora, A. Mazzone, E. Ricchiuti, C. Santini, A. Rosato, D. Tirotta, L. Giampaolo, F. Corradi, A. Torrigiani, S. Di Gregorio, R. Gerloni, L. Parodi, P. Gnerre, V. Vannucchi, F. Pallini, G. Landini, P. Giuri, G. Prampolini, D. Arioli, M. C. Leone, C. Canale, F. Condemi, R. Lupica, F. Manzola, R. Mascianà, G. Agnelli, C. Becattini, E. D’Agostini, M. G. Mosconi, G. Bogliari, A. Rossi, M. Campanini, G. Iannantuoni, L. Bartolino, A. Montagnani, V. Verdiani, M. Gambacorta, S. Lenti, S. Francioni, M. G. Pierfranceschi, C. Cattabiani, F. Orlandini, L. Scuotri, M. La Regina, F. Corsini, L. Anastasio, N. Mumoli, V. Mazzi, A. Camaiti, G. Balbi, F. Ragazzo, M. Pengo

**Affiliations:** 1 Intermediate Care Unit, Azienda Ospedaliero-Universitaria Careggi, Florence, Italy; 2 Internal Medicine, Hospital “Santa Maria Nuova” Florence, Florence, Italy; 3 Internal Medicine, P.O. “Barone Lombardo”, Canicattì, AG Italy; 4 Internal Medicine, Hospital of Luino, ASST-Sette Laghi, and University of Insubria, Varese, Italy; 5 Medical Department, Internal Medicine, Hospital “Cardarelli”, Naples, Italy; 6 Internal Medicine, Hospital of Prato, Prato, Italy; 7 Internal Medicine, Hospital “Civile” of Senigallia, Ancona, Italy; 8 Medical Department, Internal Medicine, Hospital “Civile” of Legnano, Milan, Italy; 9 Medical Department, Internal Medicine, Hospital “Vannini”, Rome, Italy; 10 Internal Medicine, Hospital of Cattolica, Rimini, Italy; 11 Medical Department, Internal Medicine 2, Azienda Ospedaliero-Universitaria Careggi, Florence, Italy; 12 Internal Medicine, “Ospedali Riuniti di Trieste”, Trieste, Italy; 13 Internal Medicine, “San Paolo” Hospital, Savona, Italy; 14 Research Department, FADOI Foundation, Piazzale Cadorna, 15, 20123 Milan, Italy; 15 Department of Internal Medicine, Hospital “Maggiore della Carità”, Novara, Italy; 16 Department of Internal Medicine, Ospedale Fatebenefratelli-AFaR, Isola Tiberina, Rome, Italy; 17 Medical Department, Hospital “Buon Consiglio-Fatebenefratelli”, Naples, Italy

**Keywords:** Community-acquired pneumonia, Cardiovascular events

## Abstract

**Background:**

The burden of cardiovascular (CV) complications in patients hospitalised for community-acquired pneumonia (CAP) is still uncertain. Available studies used different designs and different criteria to define CV complications. We assessed the cumulative incidence of acute of CV complications during hospitalisation for CAP in Internal Medicine Units (IMUs).

**Methods:**

This was a prospective study carried out in 26 IMUs, enrolling patients consecutively hospitalised for CAP. Defined CV complications were: newly diagnosed heart failure, acute coronary syndrome, new onset of supraventricular or ventricular arrhythmias, new onset hemorrhagic or ischemic stroke or transient ischemic attack. Outcome measures were: in-hospital and 30-day mortality, length of hospital stay and rate of 30-day re-hospitalisation.

**Results:**

A total of 1266 patients were enrolled, of these 23.8% experienced at least a CV event, the majority (15.5%) represented by newly diagnosed decompensated heart failure, and 75% occurring within 3 days. Female gender, a history of CV disease, and more severe pneumonia were predictors of CV events. In-hospital (12.2% vs 4.7%, *p* < 0.0001) and 30-day (16.3% vs 8.9%, *p* = 0.0001) mortality was higher in patients with CV events, as well as the re-hospitalisation rate (13.3% vs 9.3%, *p* = 0.002), and mean hospital stay was 11.4 ± 6.9 vs 9.5 ± 5.6 days (p < 0.0001). The occurrence of CV events during hospitalisation significantly increased the risk of 30-day mortality (HR 1.69, 95% CI 1.14–2.51; *p* = 0.009).

**Conclusion:**

Cardiovascular events are frequent in CAP, and their occurrence adversely affects outcome. A strict monitoring might be useful to intercept in-hospital CV complications for those patients with higher risk profile.

**Trial registration:**

NCT03798457

Registered 10 January 2019 - Retrospectively registered

**Supplementary Information:**

The online version contains supplementary material available at 10.1186/s12879-021-05781-w.

## Introduction

Community acquired pneumonia (CAP) is a frequent and clinically demanding infection [1, 2], and a leading cause of hospitalisation in Internal Medicine wards. The association between CAP and cardiovascular (CV) complications seems to be a frequent event, especially in the elderly population, and recent research has provided insights into the pathogenesis of acute CV events occurring during CAP [3–6]. Agents causing CAP can induce direct and indirect effects on the CV system. Infections induce a significant inflammatory response with the production and release of several mediators which can determine systemic effects such as activation of coagulation, enhanced platelets aggregation, and myocardial injury [7]. Mediators of the inflammatory response can modify electrophysiological properties of myocardial cells, determining increased vulnerability to cardiac arrhythmias, especially atrial fibrillation (AF) [8]. Moreover *S. pneumoniae*, the most frequent bacterial agent of CAP, determines cardiotoxicity mainly through the activity of pneumolysin, with direct consequences on myocardial injury [9, 10]. Recently, it has been suggested that CAP patients at risk for early and long-term CV events can be identified by cardiac biomarkers [11].

In the largest available studies [12, 13] roughly one-third of patients admitted for CAP suffered CV events during hospitalisation, and hospitalisation for pneumonia was associated with increased long-term risk of CV complications as well [14]. The occurrence of CV events increased significantly the overall risk of 30-day death, need for Intensive Care Unit (ICU) admission and prolonged hospital stay compared to patients not experiencing such complications. However, the burden of CV complications in CAP patients admitted to Internal Medicine Units, as well as the knowledge of predictors of such events, is still uncertain. Available studies used different designs (retrospective vs prospective), different criteria to define CV complications and were performed in heterogeneous settings such as ICU, Emergency Departments, Pneumology Units, as well as Internal Medicine and the community. Such a heterogeneity makes it difficult to retrieve reliable data regarding CAP patients hospitalised in Internal Medicine Units, which is the setting where most pneumonia patients are currently admitted.

Based on this rationale, the Italian Scientific Society of Hospital Internal Medicine (FADOI) promoted this multicentre prospective study on the Implications of acute Cardiovascular Events in patients hospitalised for Community-Acquired Pneumonia (ICECAP) in order to evaluate, in a large number of Internal Medicine wards, the prevalence and outcome of well-defined acute CV events in patients hospitalised for CAP.

## Methods

### Study design, setting and inclusion criteria

The ICECAP study was promoted and managed independently by FADOI, the Italian Scientific Society of Hospital Internal Medicine. This was a multicentre prospective study carried out in 26 Internal Medicine Units representative of the Italian territory and hospital organisation, enrolling patients consecutively hospitalised for CAP from October 2016 to February 2018. Patients were included in the study if they met the following criteria: age > 18 years, objective evidence of pneumonia defined by the presence of newly discovered abnormal infiltrates on chest radiograph or CT scan and at least two of the following clinical features consistent with pneumonia: fever > 37.8 °C, chest symptoms (dyspnea, productive cough), abnormal chest signs on physical examination (crepitation, bronchial breathing, pleural effusion), leukocytosis [15, 16]. Patients were excluded from the study if they met criteria for hospital-acquired pneumonia (HAP) (defined as any pneumonia onset after 48 h from admission or ventilator-associated pneumonia), and/or if they were severely immunocompromised (neutropenia after chemotherapy for solid or hematologic cancer, bone marrow or umbilical cord transplantation, immunosuppressive therapy for solid organ transplantation or for autoimmune diseases, HIV), or refused or were unable to give consent to participate in the study. Antimicrobial treatment was left to the discretion of the attending physician.

A sample size of 1300 patients has been calculated by hypothesising an estimated incidence of acute CV complications of 5.0 ± 1.2% (95% CI).

The study was performed in compliance with the principles of the Declaration of Helsinki on studies on human beings (Seventh revision, Fortaleza 2013). According to Italian law, a preliminary approval for the study was obtained by the Ethics Committee of the coordinating centre – Committee Hospital “Careggi” Firenze, and after that all the Ethics Committees of the participating centres gave their approval. Written informed consent was obtained from each participating patient. The study protocol has been registered at Clinical Trial.gov (NCT03798457).

### Data collection and outcome

Demographic data, clinical characteristics and laboratory values were collected at the time of hospitalisation. The CURB-65 (Confusion, Urea nitrogen, Respiratory rate, Blood pressure, and age > 65 years) and the Pneumonia Severity index (PSI) were the scores used to assess the severity of pneumonia; definition and diagnostic criteria for comorbidities are reported in the Supplementary Material. The occurrence of Acute Kidney Injury (AKI) was defined according to the Kidney Disease Improving Global Outcomes (KDIGO) criteria, as the occurrence of any of the following: increase in serum creatinine ≥0.3 mg/dl within 48 h; or increase in serum creatinine ≥1.5 times baseline, which is known or presumed to have occurred within the prior 7 days; or urine volume < 0.5 ml/kg/h for 6 h [17]. All data were registered by using a study-specific web-based case report form. Coherently with the observational nature of the study, the study protocol did not provide for diagnostic investigations to be conducted systematically in all patients, but instrumental or biohumoral examinations were performed according to the judgment of the attending physician, and with the frequency deemed clinically appropriate on a case-by-case basis.

The CV events considered during the hospitalisation and their definitions were as follows:

i- newly diagnosed heart failure: defined by the presence of typical signs and symptoms of heart failure associated with NT-proBNP values greater than 300 pg/mL in a patient without a previous history of decompensated heart failure or previous documentation of severe heart valve dysfunction and/or ventricular dysfunction (defined as left ventricular ejection fraction under 40%) as assessed by echocardiography and/or cardiac MRI and/or cardiac ventriculography and/or gated SPECT scan according to the European Society of Cardiology definition [18];
ii- acute coronary syndrome (ACS): defined by the detection of typical symptoms of ischemia associated with troponin level above the normal value (according to the reference range values of local laboratory) and/or ischemic electrocardiographic changes (new ST-T changes or new left bundle branch block) [19];
iii- new onset (i.e. documented sinus rhythm by ECG at hospital admission) atrial fibrillation or flutter;
iv- new onset ventricular arrhythmias (ventricular tachycardia, flutter or fibrillation), new onset of high degree atrio-ventricular (AV) block (2nd and 3rd);
v- new onset of haemorrhagic or ischemic stroke or transient ischemic attack (TIA) defined as a new onset of focal neurological impairment and/or the presence of haemorrhagic or ischemic lesion on brain CT/MRI scan.

Apart from CV events, information on other major clinical outcomes was collected during hospitalisation (survival, transfer to ICU for worsening of clinical conditions). Moreover, 30-day mortality and re-hospitalisation were documented. The importance of follow-up had been emphasized to the participating centers from the initial stages of the study, and the project management of the Promoter carefully followed the progression of the study work and carried out constant remote monitoring of the adequacy and completeness of the data collected. Information about survival status was obtained by telephone follow-up, or by death certificate directly available in electronic charts in use in 12 out of 26 centers, or eventually by direct information.

### Statistical analysis

The incidence of CV complications, as previously defined, was the primary evaluation parameter, and was calculated as the ratio between the observed number of patients with complications and the total number of patients at risk. A sample size of around 1300 patients has been calculated by hypothesising an incidence of acute CV complications of 5.0 ± 1.2% (95% confidence interval). This hypothesis has been defined according to preliminary data from literature, and with a conservative approach.

Continuous variables were expressed as mean ± standard deviation (SD) or median and interquartile range (IQR) values, while categorical data were expressed as proportions and percentages. The Student t-test and one-way analysis of variance (ANOVA) models were used for the comparison of continuous normally distributed variables, and the Mann-Whitney U test for continuous not normally distributed variables. The chi square test or Fisher’s exact test were used for the comparison of categorical variables. Risk factors for the occurrence of in-hospital CV events were evaluated using univariate and multivariable logistic regression analysis, and expressed as odds ratio (OR) and 95% confidence intervals (CIs). In the analysis were considered demographic and clinical variables, such as comorbidities and pneumonia severity elements (i.e. PSI, pleural effusion and multilobar infiltrates). To assess the risk of 30-day mortality univariate and multivariable Cox proportional hazards analysis were performed using a forward regression model with an entry probability set at 0.05 and removal at 0.10, for each variable. Variables included in the analysis, after evaluation for collinearity, were demographic and clinical items, such as comorbidities, PSI, and pneumonia severity features, as well as the occurrence of acute kidney injury (AKI) and CV events during hospitalisation. Curves describing the proportional hazards over time were constructed according to the Cox model. All *p*-values were two-tailed and considered significant when < 0.05 (95% CI). All analyses were performed using the Statistical Package for Social Sciences 21.0 (SPSS Inc., Chicago, Ill, USA).

## Results

### General characteristics and outcomes of the study population

**Fig. 1:**
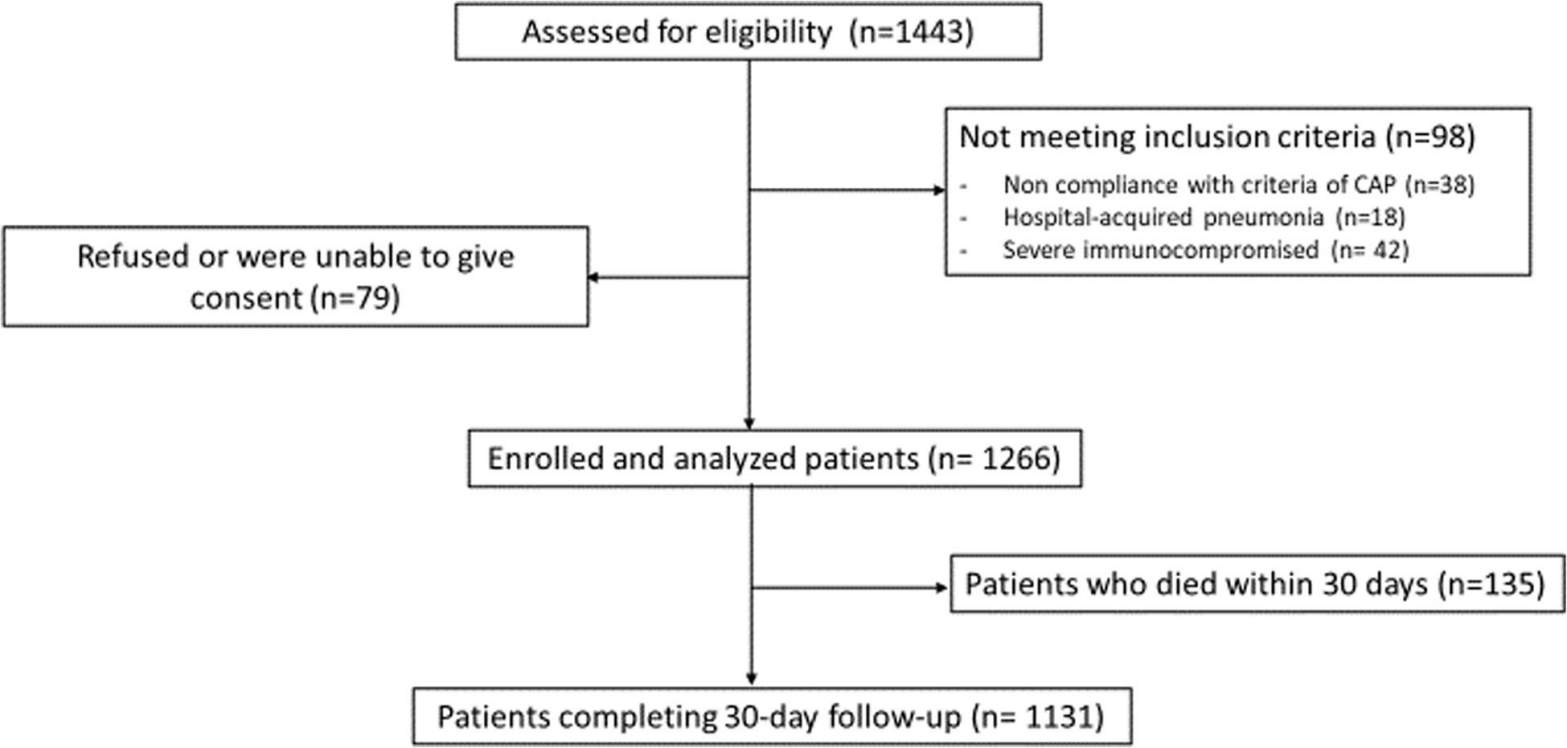
Patients’ disposition in the study

During the study period, a total of 1443 patients admitted with pneumonia were screened (Fig. 1). Of these, 1266 (87.7%) met the inclusion criteria and were eligible for the study. Most patients were elderly, with a median age of 79 years. Female and male genders were equally represented. The prevalence of comorbidities was high, with roughly one-fifth of the population having more than 3 comorbidities, mainly represented by CV diseases, chronic obstructive pulmonary disease (COPD), diabetes and moderate-severe chronic kidney disease (Table 1). Most patients had a profile of high disease severity at pneumonia severity scores; 90% of patients were in PSI class IV or V, and 28.9% were in CURB-65 class 3–5. One quarter of patients had radiological signs of high severity represented by multilobar infiltrates and/or pleural effusion (Table 2). Table 3 describes the distribution of the main categories of antibiotics used, as monotherapy or combination therapy, in the overall study population and in the subgroups of patients with or without CV events.

**Table 1:** Demographic and clinical characteristics of the general population and of patients experiencing or not experiencing CV events during hospitalisation for CAP

	General population *N* = 1266 (100%)	Cardiovascular events		***P*** value
		Yes 301 (23.8%)	No 965 (76.2%)	
**Demographic Characteristics**				
Age mean (± SD)	76.2 ± 13.8	81.2 ± 9.2	74.6 ± 14.7	0.0001
Age median (IQR)	79 (71–86)	83 (76–88)	78 (69–85)	0.0001
Age ≥ 75 years	825 (65.2%)	240 (79.7%)	585 (60.6%)	< 0.0001
Female sex	615 (48.6%)	168 (55.8%)	447 (46.3%)	0.004
Nursing home resident	61 (4.8%)	15 (5.0%)	46 (4.8%)	0.878
**Cardiovascular Risk Factors and Diseases**				
Current smoker	494 (39%)	108 (35.9%)	386 (40.0%)	0.062
Former smoker	192 (15.2%)	34 (11.3%)	158 (16.4%)	0.058
Peripheral arterial disease	82 (6.5%)	25 (8.3%)	57 (5.9%)	0.142
COPD	347 (27.4%)	84 (27.9%)	263 (27.3%)	0.825
Coronary artery disease	212 (16.7%)	73 (24.3%)	139 (14.4%)	< 0.0001
Ischemic stroke/TIA	132 (10.4%)	41 (13.6%)	91 (9.4%)	0.056
Haemorrhagic stroke	20 (1.6%)	5 (1.7%)	15 (1.6%)	1
Diabetes mellitus	300 (23.7%)	76 (25.2%)	224 (23.2%)	0.437
Dyslipidemia	156 (12.3%)	35 (11.6%)	121 (12.5%)	0.763
Venous thromboembolism	26 (2.1%)	2 (0.7%)	24 (2.5%)	0.061
Chronic liver insufficiency	20 (1.6%)	6 (2.0%)	14 (1.5%)	0.595
History of paroxysmal or permanent Atrial fibrillation/Flutter	257 (20.3%)	87 (28.9%)	170 (17.6%)	< 0.0001
Hypertension	694 (54.8%)	209 (69.4%)	485 (50.3%)	< 0.0001
CKD (GFR < 60 ml/min)	162 (12.8%)	47 (15.6%)	115 (11.9%)	0.113
Cancer	207 (16.4%)	49 (16.3%)	158 (16.4%)	1
Obesity	74 (5.8%)	20 (6.6%)	54 (5.6%)	0.484
Chronic heart failure	269 (21.3%)	82 (27.2%)	187 (19.4%)	0.005
Dementia	195 (15.4%)	47 (15.6%)	148 (15.3%)	0.927
> 3 comorbidities	246 (19.4%)	80 (26.6%)	166 (17.2%)	0.0001
**In-hospital and 30-day Outcome**				
Length of stay median days (IQR)	9 (6–12)	10 (7–13)	8 (6–11)	0.0001
In-hospital mortality	78 (6.2%)	34 (12.2%)	44 (4.7%)	< 0.0001
30-day mortality	135 (10.7%)	49 (16.3%)	86 (8.9%)	0.0001
30-day re-hospitalisation	133 (10.5%)	40 (13.3%)	90 (9.3%)	0.002
**Selected Cardiovascular drugs**				
ACE-i/ARBs	553 (43.7%)	150 (49.8%)	403 (41.8%)	0.016
Statins	289 (22.8%)	68 (22.6%)	221 (22.9%)	0.937
Beta-blockers	398 (31.4%)	138 (45.8%)	260 (26.9%)	< 0.0001
Calcium channel blockers	229 (18.1%)	50 (16.6%)	179 (18.5%)	0.493
Diuretics	545 (43%)	149 (49.5%)	396 (41.0%)	0.011
Anticoagulants	195 (15.4%)	55 (18.3%)	140 (14.5%)	0.12
Antiplatelets	455 (35.9%)	141 (46.8%)	314 (32.5%)	< 0.0001

*COPD* Chronic obstructive pulmonary disease, *CKD* Chronic kidney disease, *GFR* Glomerular filtration rate, *ACE-i* Angiotensin converting enzyme inhibitors, *ARBs* Angiotensin receptor blockers

**Table 2:** Pneumonia severity assessed by CURB-65 and PSI score

Pneumonia Severity and Scores	General population *N* = 1266 (100%)	Cardiovascular events		***P*** value
		Yes 301 (23.8%)	No 965 (76.2%)	
Pleural Effusion	320 (25.3%)	94 (31.2%)	226 (23.4%)	0.008
Multilobar infiltrates	341 (26.9%)	86 (28.6%)	255 (26.4%)	0.458
CURB-65 (mean ± SD)	2.0 ± 1.0	2.3 ± 0.9	1.9 ± 1.0	0.0001
CURB-65 ≥ 3	366 (28.9%)	114 (37.9%)	252 (26.1%)	0.0001
PSI (mean ± SD)	144.7 ± 38.0	157.2 ± 34.3	140.9 ± 38.3	0.0001
PSI class I-III (1–90 points)	108 (8.5%)	10 (3.5%)	98 (10.1%)	0.0001
PSI class IV (91–130 points)	315 (24.9%)	55 (18.3%)	260 (26.9%)	0.0001
PSI class V (> 130 points)	843 (66.6%)	236 (78.4%)	607 (62.9%)	0.0001

**Table 3:** Antibiotics used for the treatment of CAP during hospitalisation. The initial antibiotic treatment involved monotherapy or combination therapy

Antibiotic Treatment	General population	Cardiovascular event		***P*** value
	1266 (%)	Yes 301 (23.8%)	No 965 (76.2%)	
Penicillin^a^	596 (47.1)	133 (44.2)	463 (48)	0.26
Cephalosporin	405 (32)	106 (35.2)	299 (31)	0.17
Quinolone	359 (28.4)	64 (21.3)	295 (30.6)	0.002
Macrolide	317 (25)	74 (24.6)	243 (25.2)	0.87
Carbapenem	31 (2.4)	9 (3)	22 (2.3)	0.52
Other^b^	94 (7.4)	25 (8.3)	65 (6.7)	0.37

^a^Penicillin with or without beta-lactamase inhibitor

^b^Other classes of antibiotic used were imidazole, sulphamydic, tetracyclin, oxazolodinedion, alone or combined with other antibiotic classes

The mean hospital stay was 10 ± 5.9 days, the in-hospital and 30-day mortality rates were 6.2% (78 patients) and 10.7% (135 patients), respectively. Fifty-one patients (4%) were transferred to the ICU for clinical deterioration requiring mechanical ventilation and/or advanced organ support; of these, 10 (19.6%) were died at 30-day follow-up. The rate of re-hospitalisation at 30 days was 10.5% (133 patients). No patient refused or was lost to the 30-day follow-up.

### Frequency, type, and timing of CV complications

**Fig. 2:**
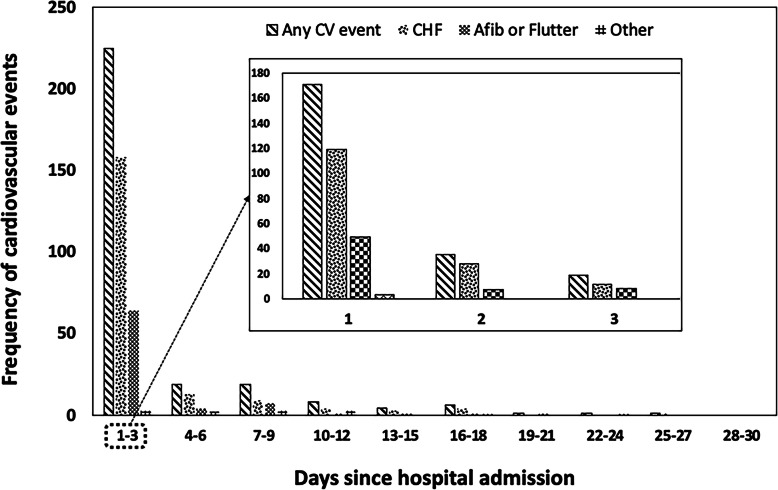
Distribution and type of cardiovascular events over time since hospital admission for CAP

During the in-hospital stay, 301 patients (23.8%) experienced at least a CV event. Of these, 196 patients (15.5%) had newly diagnosed decompensated heart failure, 111 patients (8.7%) had atrial fibrillation or flutter, 34 (2.7%) had an acute coronary syndrome, 11 (0.8%) experienced ischemic stroke or TIA. A minority of patients experienced advanced degree AV block (3 patients), ventricular tachyarrhythmias (4 patients), or hemorrhagic stroke (1 patient). Most of the CV complications occurred within the first 7 days of admission. In 75% percent of patients (225/301) the qualifying event occurred within 3 days, and in 56.8% (171/301) within the first day; the median and mean time of occurrence were 1 and 4 days, respectively. The distribution over time among the different types of events appeared similar (Fig. 2).

### Risk factors for CV complications

Risk factors for the occurrence of CV complications during hospitalisation are shown in Table 4. According to the results of both univariate and multivariable analyses, female gender and a history of CV disease were predisposing factors for an increased risk of CV complications during hospitalisation. Similarly, a higher pneumonia severity profile defined by PSI > 130 points, and the presence of pleural effusion significantly increased the risk of CV complications during hospitalisation for CAP. Moreover, the occurrence of acute kidney injury according to KDIGO criteria [17] during hospitalisation was an independent risk factor for the CV events. Risk factors for the occurrence of the most relevant CV events (newly diagnosed heart failure, new onset atrial fibrillation or flutter, acute coronary syndrome) during hospitalisation are described in detail in Tables of Supplementary Material. In general, risk factors for the single CV end-points were similar to that for the occurrence of any CV complication, and were a higher CV risk profile at medical history, more severe pneumonia and acute kidney injury during hospitalisation (Supplementary Material Tables 1, 2 and 3).

**Table 4:** Risk factors for the occurrence of any CV event during hospitalisation for CAP at univariate and multivariable logistic regression analysis

	Univariate logistic regression analysis			Multivariable logistic regression analysis		
	OR	(95% CI)	***p***	OR	(95% CI)	***p***
Sex (female)	1.47	1.12–1.63	0.004	1.72	1.30–2.27	0.0001
COPD	1.00	0.77–1.38	0.82			
CAD	1.90	1.38–2.62	0.0001	1.95	1.39–2.74	0.0001
Stroke/TIA	1.58	0.96–2.51	0.055			
Diabetes mellitus	1.13	0.84–1.53	0.44			
Arterial Hypertension	2.25	1.71–2.96	0.0001	2.02	1.52–2.69	0.0001
Moderate-Severe CKD (GFR < 60 ml/min)	1.37	0.95–1.97	0.11			
Heart failure	1.56	1.15–2.10	0.004			
Dementia	1.02	0.71–1.46	0.92			
Comorbidities> 3	1.74	1.28–2.36	0.0001			
Pleural effusion	1.48	1.12–1.98	0.008	1.39	1.03–1.88	0.028
Multilobar pneumonia	1.11	0.83–1.49	0.46			
Acute Kidney Injury during hospitalisation	2.07	1.50–2.87	0.0001	1.66	1.18–2.35	0.003
PSI > 130 points (Class V)	2.14	1.58–2.90	0.0001	1.79	1.30–2.47	0.0001

*COPD* Chronic obstructive pulmonary disease, *CAD* Coronary artery disease, *TIA* Transient ischemic attack, *CKD* Chronic kidney disease, *GFR* Glomerular filtration rate

### Relationship between CV events and in-hospital and 30-day outcome

**Fig. 3:**
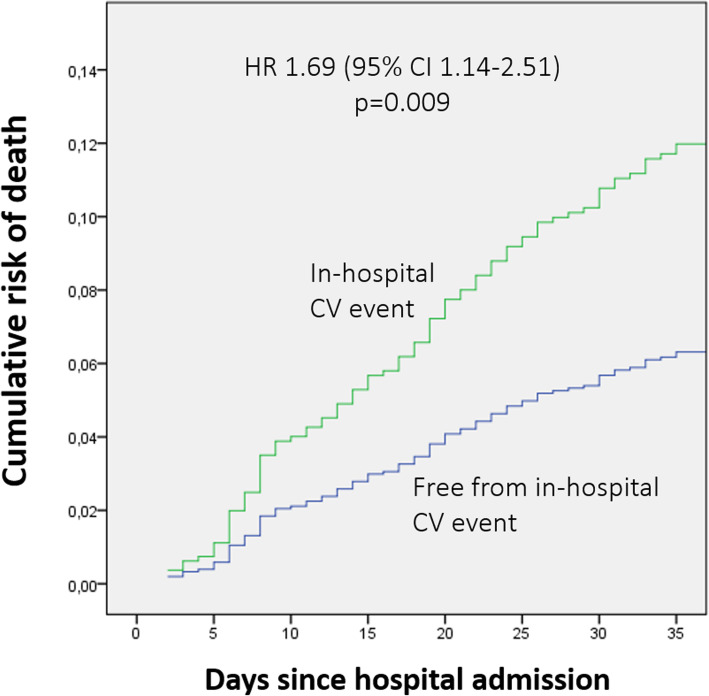
Estimated risk of 30-day mortality by Cox proportional hazard model of CAP patients according to the presence or absence of CV events during hospitalisation

Patients who experienced CV events during hospitalisation had higher in-hospital and 30-day mortality with respect to those free from events. Specifically, in-hospital deaths occurred in 34 (12.2%) patients with CV events, and in 44 (4.7%) patients free from CV events (*p* < 0.0001); 30-day deaths occurred in 49 patients (16.3%) with and in 86 patients (8.9%) without CV events, respectively (*p* = 0.0001). Similarly, length of hospital stay and rate of re-hospitalisation within 30 days were higher for those patients who experienced CV events with respect to those event-free; mean hospital stay was 11.4 ± 6.9 vs 9.5 ± 5.6 days (p < 0.0001), and rate of re-hospitalisation was 13.3 and 9.3% (*p* = 0.002), respectively. Cox proportional hazards analyses of selected variables for 30-day mortality are shown in Table 5. The occurrence of any CV event during hospitalisation independently and significantly increased the risk of 30-day mortality (HR 1.69, 95% CI 1.14–2.51; *p* = 0.009) when adjusted for other variables including pneumonia severity, and comorbidities (Table 5 and Fig. 3). When considered separately, newly diagnosed heart failure, new onset atrial fibrillation or flutter, acute coronary syndrome, were not associated with increased risk of a death at 30 days (Table 5).

**Table 5:** Risk factors for 30-day mortality according to univariate and multivariable Cox proportional hazards analysis

	Univariate Cox regression analysis			Multivariable Cox regression analysis		
	HR	95%CI	***p***	HR	95%CI	***p***
Female gender	1.06	0.76–1.46	0.78			
Multilobar infiltrates	1.25	0.85–1.85	0.458			
Pleural effusion	1.28	0.86–1.89	0.25			
PSI > 130 points (Class V)	3.88	2.30–6.55	0.0001	2.87	1.64–5.02	0.0001
Any in-hospital CV event	1.99	1.36–2.90	0.001	1.69	1.14–2.51	0.009
Newly diagnosed decompensated heart failure	1.62	1.56–2.55	0.039			
New onset AF or flutter	1.04	0.64–1.58	0.99			
CAD	1.02	0.64–1.65	0.9			
COPD	1.04	0.71–1.55	0.84			
Stroke/TIA	1.71	0.74–3.93	0.20			
Arterial Hypertension	0.77	0.54–1.09	0.145			
Cancer	1.69	1.10–2.60	0.019			
Dementia	2.89	1.93–4.31	0.0001	2.41	1.58–3.67	0.0001
Comorbidities > 3	1.74	1.16–2.60	0.011			
Acute Kidney Injury during hospitalisation	2.31	1.53–3.49	0.0001	1.86	1.21–2.87	0.005
CKD	1.84	1.16–2.92	0.013			

*AF* Atrial fibrillation, *CAD* Coronary artery disease, *COPD* Chronic obstructive pulmonary disease, *TIA* Transient ischemic attack, *CKD* Chronic kidney disease

## Discussion

In this large multicentre trial, designed to investigate the prevalence and implications of CV events in patients admitted for CAP to Internal Medicine wards in Italy (ICECAP), we observed a high prevalence of in-hospital CV complications with a significant impact on in-hospital and 30-day outcome.

The prevalence of in-hospital CV events in this study was 23.8%; generally speaking this means that approximately one out of four patients admitted for CAP is expected to suffer from a CV event during hospitalisation for pneumonia. This result confirms previous observations, where a prevalence of CV events during hospitalisation for pneumonia has been reported in the range of 27 to 32.2% [12, 13].

Most of the vascular events detected in our study were of cardiac origin, mainly represented by newly diagnosed decompensated heart failure (15.5%), atrial fibrillation or flutter (8.7%), and acute coronary syndromes (2.7%), while cerebral events occurred in a minority of patients (i.e. less than 1%), as well as the occurrence of advanced degree AV block and ventricular arrhythmias.

Some differences between ICECAP and the two above-mentioned studies exist and should be emphasised. Data from the landmark study by Corrales-Medina et al. [12], referred to a cohort of hospitalised and out-patients dating back to the early 1990’s when troponin and natriuretic peptides were not available, and then not in use for the definition of CV endpoints; therefore, the results could not reflect the current prevalence and significance of CV events occurring during hospitalisation for CAP. The SIXTUS study [13], which is a contemporary and well-designed trial, involved only three hospitals in Italy and Canada. In this perspective, the study population could have some limitations for the generalisability of the observed results.

A significant difference between ICECAP and previous studies addressing the association between CV events and CAP is the definition of some CV endpoints. In ICECAP we used an objective definition of newly diagnosed heart failure that included clinical, instrumental and biohumoral confirmation with NT-proBNP measurement. The difference in definition, with a more strict use of heart failure criteria, is probably the main reason for the lower prevalence of heart failure as a CAP complication in the ICECAP (15.5%) with respect to other studies (20.8 to 23.8%) [12, 13]. Similarly, we decided to consider only new episodes of supraventricular arrhythmias (atrial fibrillation, atrial flutter), and not worsening arrhythmias as considered in previous studies [12, 20]. The decision to use a stricter definition of these endpoints was done in order to identify only those CV events directly related to pneumonia.

The large majority of CV events occurred early during hospitalisation. Specifically, more than half of events occurred within the first 24 h of admission, and 76.8% occurred within 3 days. These results are in line with previous findings, because in the cohort by Corrales-Medina et al. and in the SIXTUS study, CV events occurred within the first day in 55% of patients and within 2 days in 61% of cases, respectively [12, 13]. In our study, the vast majority of CV events occurred within the first 3 days of hospitalisation for CAP, and special attention should be focused in this time window in order to intercept CV complications. This is particularly true for those patients who appeared at higher risk such as those over the age of 75, with a history of coronary artery disease, arterial hypertension, and more severe pneumonia (PSI > 130, pleural effusion). These observations are not unexpected, as all these elements correlate with a higher CV burden and risk of complications, as supported by biological plausibility and results from other studies [12, 13, 21, 22].

Interestingly, in ICECAP the occurrence of AKI during hospitalisation was an independent risk factor for the occurrence of CV events. We can speculate that AKI occurring in the context of CAP represents two sides of the same coin. On one side the occurrence of AKI can be the expression of a more severe inflammatory burden. In fact, it has been shown that elevated and imbalanced pro- and anti-inflammatory mediators along with severe endothelial dysfunction and ischemia may induce kidney injury [23]. On the other side, acute renal injury per se determines detrimental effects increasing the risk of CV events [17].

It has been reported that some types of antibiotic treatment can influence the outcome and CV risk in patients hospitalised for pneumonia [24]. In particular, it was suggested that quinolones could increase the risk of arrhythmias [25, 26], but this was not the case in our experience. Indeed, in our study the treatment with quinolones was found to be inversely related to the occurrence of CV events. In our opinion this result is difficult to interpret for several reasons, the more relevant probably being the use of quinolones in association with other antibiotics, and the influence of fluid and electrolyte balance on the occurrence of CV events. All these factors could have affected the occurrence of CV events independently from quinolone use, and finally, we cannot exclude that this observation depends on chance even in the light of what is present in literature and providing neutral or opposite indications.

It is well known that CAP is associated with a relevant risk of mortality during hospital stay and afterwards [27], and previous findings have suggested that CV complications are associated with poor outcome in community-acquired pneumonia [28, 29]. According to the results of our study, the occurrence of CV events in people admitted for CAP has negative consequences on in-hospital and 30-day outcome. The absolute in-hospital and 30-day mortality of patients with CV events were particularly high with respect to those free of events; 12.2% vs 4.7, and 16.3% vs 8.9%, respectively. Remarkably, the occurrence of CV events independently and significantly increased the risk of death at 30 days (HR 1.69, 95% CI 1.14–2.51; *p* = 0.009) when adjusted for other variables. Only the development of AKI during hospitalisation, dementia, and PSI > 130 points were associated with a substantial risk increase in 30-day mortality. These findings were similar to those reported in the study by Corrales-Medina et al., where the occurrence of a CV event independently increased the probability of 30-day death by a factor of 1.7. These results differ substantially from those reported in the SIXTUS study, where the risk of 30-day mortality attributed to a CV event during hospitalisation increased nearly five-fold.

In addition, CV complications increased significantly the risk of having a longer hospital stay as well as re-hospitalisation at 30 days, with respect to those patients with an uncomplicated course. In the case of occurrence of a CV event, the length of hospital stay increased significantly by an average of 2 days (median 10 days [IQR 7–13] vs 8 days [IQR 6–11]; *p* = 0.0001), whilst the probability of being readmitted to hospital in the following month increased by a factor of 1.6 (HR 1.62, 95% CI 1.09–2.42; *p* = 0.02).

A possible limitation of this study can be the lack of standardised management of CAP in the context of participating centres, and the generalisability of observations to other countries where different healthcare organisations (with reference to the role of Internal Medicine) are in place. A second limitation of the study is the exclusion of antimicrobial treatments from statistical analysis of outcome measures. After several considerations, we decided to avoid the inclusion of such variables in regression models. Gathering information for antimicrobial treatment is complex, as an example a single patient can receive multiple antibiotics at different times during the in-hospital course for different reasons, thus obtaining sound information from spurious data can be difficult and possibly misleading. Similarly, another limitation can be that cardiovascular medications were tested as variables at univariate analysis, but not considered for the multivariable model. The reasons supporting this decision were that cardiovascular drugs were registered on admission and prescribed for conditions prior to the hospitalisation for CAP. At univariate analysis, drugs such as ACE-i/ARBs, Beta-blockers and antiplatelets were frequently associated with an increased risk of CV events during hospitalisation for CAP (Supplementary Material Tables 1, 2 and 3). These results seem in contrast with biological plausibility and well-documented scientific evidence proving a protective CV effect of ACE-i/ARBs, Beta-blockers, statins and antiplatelets in many clinical conditions (i.e. surgery, secondary prevention, etc). Indeed, the results of univariate analysis very likely reflect the greater prevalence of CV medications in patients with a history of cardiovascular diseases. In view of these considerations, many potential confounders related to cardiovascular drugs could have induced a bias in the analysis, interpretation and reliability of results, thus we consciously decided not to include cardiovascular medications in the multivariable model.

On the other hand, ICECAP is the largest and contemporary multicentre study offering a comprehensive overview on the prevalence and clinical significance of CV events occurring during hospitalisation for CAP in Internal Medicine Units. The considerable number of centres involved, the clear definition of CV endpoints, and the significant number of patients enrolled at each centre, provide sound results which substantially integrate current evidence in this field and suggest possible practical implications.

In view of the results of this study, which confirm recent observations on the burden of CV complications during hospitalisation for pneumonia, CV events should be regarded as a common and unfavourable complication of CAP. For these reasons, they should be considered together with more classical but less frequent complications, such as pleural empyema and pulmonary abscesses, as common events in the course of pneumonia disease. Direct ECG monitoring or telemetry during the first 3–4 days of admission, in selected patients with a higher baseline CV risk profile (i.e. older age, high baseline CV burden, more severe pneumonia), could be an appropriate strategy to intercept early CV complications that can have detrimental impact on the short- and long-term outcome.

### Supplementary Information

**Additional file 1.**

## Data Availability

The data sets compiled and analyzed for the current study are available from the corresponding author on reasonable request.

## Appendix

We would like to mention all the members of the ICECAP Study Group who actively contributed in the enrolment of patients and data collection (listed per centre and in order of number of enrolled patients):

T. Attardo, G. Augello, (Canicattì – AG); F. Dentali, L. Tavecchia, V. Gessi (Varese); F. Pieralli, G. De Marzi, A. Torrigiani, L. Corbo (Firenze); G. Uomo, F. Gallucci, C. Mastrobuoni, F. (Napoli); F. Risaliti, A. Giani (Prato); L. Morbidoni, Consalvo Teodora (Senigallia – AN); A. Mazzone, E. Ricchiuti (Legnano -MI); C. Santini, A. Rosato (Roma); D. Tirotta, L. Giampaolo (Cattolica - RN); F. Corradi, A. Torrigiani, S. Di Gregorio (Firenze); R. Gerloni (Trieste); L. Parodi, P. Gnerre (Savona); V. Vannucchi, F. Pallini, G. Landini, (Firenze); P. Giuri, G. Prampolini (Castelnuovo nei Monti – RE); D. Arioli, MC Leone (Reggio Emilia); C. Canale, F. Condemi, R. Lupica, F. Manzola, R. Mascianà (Reggio Calabria); G. Agnelli, C. Becattini, E. D’Agostini, MG Mosconi, G. Bogliari (Perugia); A. Rossi, M. Campanini, G. Iannantuoni, L. Bartolino (Novara); A. Montagnani, V. Verdiani (Grosseto); M. Gambacorta (Pantalla- Todi – PG); S. Lenti, S. Francioni (Arezzo); MG Pierfranceschi, C. Cattabiani (Fiorenzuola D’Arda – PC); F. Orlandini, L. Scuotri, M. La Regina, F. Corsini (La Spezia); L. Anastasio (Vibo Valentia); N. Mumoli, V. Mazzi, A. Camaiti (Livorno); G. Balbi, F. Ragazzo, M. Pengo (Vicenza).

## Abbreviations

ACS: Acute coronary syndrome
AKI: Acute kidney injury
ANOVA: One-way analysis of variance
CAP: Community acquired pneumonia
CIs: Confidence intervals
COPD: Chronic obstructive pulmonary disease
CURB-65 score: Confusion, Urea nitrogen, Respiratory rate, Blood pressure, and age > 65 years
CV: Cardiovascular
FADOI: Italian Scientific Society of Hospital Internal Medicine
HAP: Hospital-acquired pneumonia
ICECAP: Implications of acute Cardiovascular Events in patients hospitalised for Community-Acquired Pneumonia
ICU: Intensive Care Unit
IMUs: Internal Medicine Units
IQR: Median and interquartile range
OR: Odds ratio
PSI: Pneumonia Severity Index
SD: Standard deviation
TIA: Transient ischemic attack

## References

[1] Prina E, Ranzani OT, Torres A (2015). Community-acquired pneumonia. Lancet.

[2] File TM, Marrie TJ (2010). Burden of community-acquired pneumonia in north American adults. Postgrad Med.

[3] Restrepo MI, Reyes LF, Anzueto A (2016). Complication of community-acquired pneumonia (including cardiac complications). Semin Respir Crit Care Med.

[4] Restrepo MI, Reyes LF (2018). Pneumonia as a cardiovascular disease. Respirology.

[5] Feldman C, Normark S, Henriques-Normark B, Anderson R (2019). Pathogenesis and prevention of risk of cardiovascular events in patients with pneumococcal community-acquired pneumonia. J Intern Med.

[6] Tralhão A, Póvoa P (2020). Cardiovascular events after community-acquired pneumonia: a global perspective with systematic review and meta-analysis of observational studies. J Clin Med.

[7] Violi F, Carnevale R, Calvieri C (2015). Nox2 up-regulation is associated with an enhanced risk of atrial fibrillation in patients with pneumonia. Thorax.

[8] Hu YF, Chen YJ, Lin YJ, Chen SA (2015). Inflammation and the pathogenesis of atrial fibrillation. Nat Rev Cardiol.

[9] Anderson R, Nel JG, Feldman C (2018). Multifaceted role of pneumolysin in the pathogenesis of myocardial injury in community-acquired pneumonia. Int J Mol Sci.

[10] Shenoy AT, Beno SM, Brissac T, Bell JW, Novak L, Orihuela CJ (2018). Severity and properties of cardiac damage caused by Streptococcus pneumoniae are strain dependent. PLoS One.

[11] Menéndez R, Méndez R, Aldás I (2019). Community-acquired pneumonia patients at risk for early and long-term cardiovascular events are identified by cardiac biomarkers. Chest.

[12] Corrales-Medina VF, Musher DM, Wells GA, Chirinos JA, Chen L, Fine MJ (2012). Cardiac complications in patients with community-acquired pneumonia: incidence, timing, risk factors, and association with short-term mortality. Circulation.

[13] Violi F, Cangemi R, Falcone M (2017). Cardiovascular complications and short-term mortality risk in community-acquired pneumonia. Clin Infect Dis.

[14] Corrales-Medina VF, Alvarez KN, Weissfeld LA, Weissfeld LA (2015). Association between hospitalization for pneumonia and subsequent risk of cardiovascular disease. JAMA.

[15] Lim SW, Baudouin SV, George RC (2009). Pneumonia guidelines committee of the BTS standards of care committee. British Thoracic Society guidelines for the management of community acquired pneumonia in adults: update 2009. Thorax.

[16] Wunderink RG, Waterer GW (2014). Community-acquired pneumonia. N Engl J Med.

[17] Kidney Disease: Improving Global Outcomes (KDIGO) Acute Kidney Injury Work Group (2012). KDIGO clinical practice guideline for acute kidney injury. Kidney Int Suppl.

[18] Ponikowski P, Voors AA, Anker SD (2016). 2016 ESC guidelines for the diagnosis and treatment of acute and chronic heart failure the task force for the diagnosis and treatment of acute and chronic heart failure of the European Society of Cardiology (ESC). Eur Heart J.

[19] Roffi M, Patrono C, Collet JP (2016). 2015 ESC guidelines for the management of acute coronary syndromes in patients presenting without persistent ST-segment elevation: task force for the Management of Acute Coronary Syndromes in Patients Presenting without Persistent ST-Segment Elevation of the European Society of Cardiology (ESC). Eur Heart J.

[20] Cilli A, Cakin O, Aksoy E (2018). Acute cardiac events in severe community-acquired pneumonia: a multicenter study. Clin Respir J.

[21] Corrales-Medina VF, Suh KN, Rose G (2011). Cardiac complications in patients with community-acquired pneumonia: a systematic review and meta-analysis of observational studies. PLoS Med.

[22] Aliberti S, Ramirez JA (2014). Cardiac diseases complicating community acquired pneumonia. Curr Opin Infect Dis.

[23] Ronco C, Kellum JA, Bellomo R, House AA (2008). Potential interventions in sepsis-related acute kidney Iinjury. Clin J Am Soc Nephrol.

[24] Mortensen EM, Halm EA, Pugh MJ (2014). Association of azithromycin with mortality and cardiovascular events among older patients hospitalized with pneumonia. JAMA.

[25] van der Hooft CS, Heeringa J, van Herpen G, Kors JA, Kingma JH, Stricker BH (2004). Drug-induced atrial fibrillation. J Am Coll Cardiol.

[26] Bolognesi M, Bolognesi D (2014). Ciprofloxacin-induced paroxysmal atrial fibrillation. OA Case Rep.

[27] Yende S, D’Angelo G, Kellum JA, for the GenIMS Investigators (2008). Inflammatory markers at hospital discharge predict subsequent mortality after pneumonia and sepsis. Am J Respir Crit Care Med.

[28] Mandal P, Chalmers JD, Choudhury G, Akram AR, Hill AT (2011). Vascular complications are associated with poor outcome in community-acquired pneumonia. QJM.

[29] Bosch NA, Cohen DM, Walkey AJ (2019). Risk factors for new-onset atrial fibrillation in patients with sepsis: a systematic review and meta-analysis. Crit Care Med.

