# Correction to: Acute cardiovascular events in patients with community acquired pneumonia: results from the observational prospective FADOI-ICECAP study

**DOI:** 10.1186/s12879-021-05891-5

**Published:** 2021-02-19

**Authors:** Filippo Pieralli, Vieri Vannucchi, Carlo Nozzoli, Giuseppe Augello, Francesco Dentali, Giulia De Marzi, Generoso Uomo, Filippo Risaliti, Laura Morbidoni, Antonino Mazzone, Claudio Santini, Daniela Tirotta, Francesco Corradi, Riccardo Gerloni, Paola Gnerre, Gualberto Gussoni, Antonella Valerio, Mauro Campanini, Dario Manfellotto, Andrea Fontanella, for the FADOI-ICECAP Study Group

**Affiliations:** 1 Intermediate Care Unit, Azienda Ospedaliero-Universitaria Careggi, Florence, Italy; 2 Internal Medicine, Hospital “Santa Maria Nuova” Florence, Florence, Italy; 3 Internal Medicine, P.O. “Barone Lombardo”, Canicattì, AG Italy; 4 Internal Medicine, Hospital of Luino, ASST-Sette Laghi, and University of Insubria, Varese, Italy; 5 Medical Department, Internal Medicine, Hospital “Cardarelli”,Pieralli et al. BMC Infectious Diseases (2021) 21:116 Page 11 of 12, Naples, Italy; 6 Internal Medicine, Hospital of Prato, Prato, Italy; 7 Internal Medicine, Hospital “Civile” of Senigallia, Ancona, Italy; 8 Medical Department, Internal Medicine, Hospital “Civile” of Legnano, Milan, Italy; 9 Medical Department, Internal Medicine, Hospital “Vannini”, Rome, Italy; 10 Internal Medicine, Hospital of Cattolica, Rimini, Italy; 11 Medical Department, Internal Medicine 2, Azienda Ospedaliero-Universitaria Careggi, Florence, Italy; 12 Internal Medicine, “Ospedali Riuniti di Trieste”, Trieste, Italy; 13 Internal Medicine, “San Paolo” Hospital, Savona, Italy; 14 Research Department, FADOI Foundation, Piazzale Cadorna, 15, 20123 Milan, Italy; 15 Department of Internal Medicine, Hospital “Maggiore della Carità”, Novara, Italy; 16 Department of Internal Medicine, Ospedale Fatebenefratelli-AFaR, Isola Tiberina, Rome, Italy; 17 Medical Department, Hospital “Buon Consiglio-Fatebenefratelli”, Naples, Italy

**Correction to: BMC Infect Dis (2021) 21:116**

**https://doi.org/10.1186/s12879-021-05781-w**

After publication of the original article [1], an error was identified in one of the ICECAP Study Group member’s name, listed in the Appendix section.

The incorrect name is: MG Pierfranceschi

The correct name should be: M Giorgio-Pierfranceschi

The original article has been corrected.
