# Supplementary material for: Acute cardiovascular events in patients with community acquired pneumonia: results from the observational prospective FADOI-ICECAP study
Source: BMC Infect Dis. 2021 Jan 25;21:116. doi: 10.1186/s12879-021-05781-w (PMC7830042; doi:10.1186/s12879-021-05781-w)
Supplement: Supplementary file 1 — Additional file 1. [file 12879_2021_5781_MOESM1_ESM.docx]

**SUPPLEMENTARY MATERIALS**

- Supplementary Material Table 1. Risk factors for the occurrence of newly diagnosed heart failure during hospitalisation for CAP at univariate and multivariable logistic regression analyses
- Supplementary Material Table 2. Risk factors for the occurrence of new onset atrial fibrillation or flutter during hospitalisation for CAP at univariate and multivariable logistic regression analyses
- Supplementary Material Table 3. Risk factors for the occurrence of acute coronary syndrome during hospitalisation for CAP at univariate and multivariable logistic regression analyses
- Supplementary Material. Definition and diagnostic criteria for comorbidities

|  | **Univariate logistic regression analysis** | | | **Multivariable logistic regression analysis** | | |
| --- | --- | --- | --- | --- | --- | --- |
|  | **OR** | **(95% CI)** | **p** | **OR** | **(95% CI)** | **p** |
| Sex (female) | 0.83 | 0.61-1.13 | 0.244 |  |  |  |
| COPD | 0.98 | 0.69-1.38 | 0.931 |  |  |  |
| CAD | 1.85 | 1.29-2.67 | 0.0001 | 1.65 | 1.13-2.40 | 0.009 |
| Stroke/TIA | 1.86 | 1.12-3.09 | 0.022 |  |  |  |
| Diabetes mellitus | 0.99 | 0.69-1.42 | 1 |  |  |  |
| Arterial Hypertension | 2.19 | 1.58-3.05 | 0.0001 | 1.94 | 1.39-2.72 | 0.0001 |
| Moderate-Severe CKD (GFR< 60 ml/min) | 1.61 | 1.07-2.43 | 0.027 |  |  |  |
| Heart failure | 1.42 | 0.79-2.56 | 0.247 |  |  |  |
| Dementia | 1.04 | 0.68-1.57 | 0.830 |  |  |  |
| Comorbidities>3 | 1.74 | 1.22-2.46 | 0.003 |  |  |  |
| ACE-i/ARBs* | 1.19 | 0.88-1.62 | 0.273 |  |  |  |
| Beta-blockers* | 2.05 | 1.50-2.79 | <0.0001 |  |  |  |
| Statins* | 1.00 | 0.70-1.45 | 1 |  |  |  |
| Anticoagulants* | 1.91 | 1.31-2.77 | 0.001 |  |  |  |
| Antiplatelets* | 1.85 | 1.36-2.52 | <0.0001 |  |  |  |
| Pleural effusion | 1.63 | 1.17-2.26 | 0.004 | 1.56 | 1.11-2.18 | 0.011 |
| Multilobar pneumonia | 1.13 | 0.81-1.59 | 0.484 |  |  |  |
| Acute Kidney Injury during hospitalisation | 2.40 | 1.67-3.44 | 0.0001 | 1.85 | 1.27-2.69 | 0.001 |
| PSI > 130 points (Class V) | 2.61 | 1.77-3.84 | 0.0001 | 2.01 | 1.34-2.98 | 0.001 |

**Supplementary Material Table 1.** Risk factors for the occurrence of newly diagnosed heart failure during hospitalisation for CAP at univariate and multivariable logistic regression analyses. COPD= chronic obstructive pulmonary disease; CAD= coronary artery disease; TIA= transient ischemic attack; CKD= chronic kidney disease; GFR= glomerular filtration rate. *These variables were not included in the multivariable analysis.

|  | **Univariate logistic regression analysis** | | | **Multivariable logistic regression analysis** | | |
| --- | --- | --- | --- | --- | --- | --- |
|  | **OR** | **(95% CI)** | **p** | **OR** | **(95% CI)** | **p** |
| Sex (female) | 0.56 | 0.37-0.82 | 0.004 | 0.57 | 0.38-0.85 | 0.007 |
| COPD | 1.04 | 0.97-1.04 | 0.824 |  |  |  |
| CAD | 1.04 | 0.62-1.75 | 0.894 |  |  |  |
| Stroke/TIA | 1.03 | 0.48-2.18 | 0.848 |  |  |  |
| Diabetes mellitus | 1.00 | 0.64-1-59 | 1 |  |  |  |
| Arterial Hypertension | 1.95 | 1.28-2.96 | 0.002 | 1.91 | 1.26-2.92 | 0.003 |
| Moderate-Severe CKD (GFR< 60 ml/min) | 0.74 | 0.39-1.41 | 0.455 |  |  |  |
| Heart failure | 0.64 | 0.23-1.80 | 0.51 |  |  |  |
| Dementia | 1.33 | 0.81-2.20 | 0.269 |  |  |  |
| Comorbidities>3 | 0.98 | 0.59-1.60 | 1 |  |  |  |
| ACE-i/ARBs* | 1.27 | 0.86-1.89 | 0.268 |  |  |  |
| Beta-blockers* | 1.78 | 1.20-2.65 | 0.005 |  |  |  |
| Statins* | 0.73 | 0.44-1.21 | 0.284 |  |  |  |
| Anticoagulants* | 0.72 | 0.39-1.31 | 0.333 |  |  |  |
| Antiplatelets* | 1.16 | 0.78-1.73 | 0.469 |  |  |  |
| Pleural effusion | 1.71 | 1.13-2.58 | 0.015 | 1.66 | 1.09-2.52 | 0.017 |
| Multilobar pneumonia | 1.12 | 0.73-1.73 | 0.576 |  |  |  |
| Acute Kidney Injury during hospitalisation | 1.41 | 0.86-2.31 | 0.169 |  |  |  |
| PSI > 130 points (Class V) | 1.44 | 0.93-2.25 | 0.113 |  |  |  |

**Supplementary Material Table 2.** Risk factors for the occurrence of new onset atrial fibrillation or flutter during hospitalisation for CAP at univariate and multivariable logistic regression analyses. COPD= chronic obstructive pulmonary disease; CAD= coronary artery disease; TIA= transient ischemic attack; CKD= chronic kidney disease; GFR= glomerular filtration rate. *These variables were not included in the multivariable analysis.

|  | **Univariate logistic regression analysis** | | | **Multivariable logistic regression analysis** | | |
| --- | --- | --- | --- | --- | --- | --- |
|  | **OR** | **(95% CI)** | **p** | **OR** | **(95% CI)** | **p** |
| Sex (female) | 1.92 | 0.89-4.13 | 0.099 |  |  |  |
| COPD | 0.66 | 0.27-1.62 | 0.414 |  |  |  |
| CAD | 6.06 | 2.91-12.62 | 0.0001 | 5.39 | 2.56-11-32 | <0.0001 |
| Stroke/TIA | 0.93 | 0.22-3-98 | 1 |  |  |  |
| Diabetes mellitus | 2.21 | 1.05-4.65 | 0.047 |  |  |  |
| Arterial Hypertension | 1.24 | 0.59-2.60 | 0.584 |  |  |  |
| Moderate-Severe CKD (GFR< 60 ml/min) | 1.37 | 0.52-3.64 | 0.576 |  |  |  |
| Heart failure | 2.56 | 0.87-7.55 | 0.093 |  |  |  |
| Dementia | 1.38 | 0.56-3.43 | 0.446 |  |  |  |
| Comorbidities>3 | 2.12 | 0.98-4.59 | 0.061 |  |  |  |
| ACE-i/ARBs* | 1.71 | 0.82-3.55 | 0.192 |  |  |  |
| Beta-blockers* | 2.55 | 1.23-5.29 | 0.015 |  |  |  |
| Statins* | 1.03 | 0.44-2.42 | 1 |  |  |  |
| Anticoagulants* | 0.84 | 0.29-2.44 | 1 |  |  |  |
| Antiplatelets* | 5.10 | 2.25-11.51 | <0.0001 |  |  |  |
| Pleural effusion | 0.90 | 0.38-2.11 | 1 |  |  |  |
| Multilobar pneumonia | 1.37 | 0.63-2.95 | 0.411 |  |  |  |
| Acute Kidney Injury during hospitalisation | 3.81 | 1.81-8.04 | 0.001 | 3.14 | 1.46-6.75 | 0.003 |
| PSI > 130 points (Class V) | 1.67 | 0.71-3.92 | 0.327 |  |  |  |

**Supplementary Material Table 3.** Risk factors for the occurrence of acute coronary syndrome during hospitalisation for CAP at univariate and multivariable logistic regression analyses. COPD= chronic obstructive pulmonary disease; CAD= coronary artery disease; TIA= transient ischemic attack; CKD= chronic kidney disease; GFR= glomerular filtration rate. *These variables were not included in the multivariable analysis.

**Supplementary Material.** Definition and diagnostic criteria for comorbidities.

Comorbidities were defined by the following criteria:

Arterial hypertension: history of high blood pressure (systolic >140 mmHg and/or diastolic > 90 mmHg) and/or treatment with any antihypertensive drug with the purpose to treat hypertension.

Cancer: solid or hematological active cancer.

Chronic heart failure: history of structural and/or functional cardiac abnormalities and signs or symptoms of congestion.

Chronic kidney disease was defined by glomerular filtration (eGFR) rate value estimated by the Modification of Diet in Renal Disease (MDRD) Study equation ≤ 90ml/min. Moderate-severe chronic kidney disease was defined by eGFR value ≤ 60ml/min.

Chronic liver insufficiency: objective diagnosis of liver cirrhosis and/or presence of signs and symptoms of decompensated chirrosis

Chronic Obstructive Pulmonary disease (COPD): persistent airflow limitation during stable clinical disease at spirometry (FEV_1_/FVC <70%) and/or history of recurrent exacerbations requiring hospitalisation and/or active treatment with inhaled bronchodilators.

Dementia: reported multiple cognitive deficits in addition to memory impairment.

Diabetes mellitus: persistent hyperglycemia with glycosylated haemoglobin ≥ 48 mmol/mol and/or active treatment with insulin or any other antidiabetic drug..

Dyslipidemia: total cholesterol ≥ 240mg/dL, or trygliceride ≥ 150 mg/dL, or LDLc ≥ 140mg/dL, or HDLc ≤ 40mg/dL and/or the use of a lipid lowering drug.

Obesity: Body Mass Index (BMI) ≥ 30 Kg/m^2^.

Peripheral arterial disease (PAD): claudicatio intermittens and/or ankle-brachial index < 0,9 and/or presence of carotid atheroma on vascular ultrasound determining a reduction greater than 50% of the vessel’s lumen.

Venous thromboembolism (VTE): Previous diagnosis of deep vein thrombosis and/or pulmonary embolism.
